# Visualization of Vulnerable Coronary Plaque and Prevention of Plaque Rupture

**DOI:** 10.14789/jmj.JMJ24-0011-R

**Published:** 2024-06-15

**Authors:** TATSUYA FUKASE, TOMOTAKA DOHI

**Affiliations:** 1 Department of Cardiovascular Biology and Medicine, Juntendo University Graduate School of Medicine, Tokyo, Japan; 1 Department of Cardiovascular Biology and Medicine, Juntendo University Graduate School of Medicine, Tokyo, Japan

**Keywords:** vulnerable plaques, plaque rupture, near-infrared spectroscopy, intravascular ultrasound, plaque sealing

## Abstract

In daily clinical practice, assessing anatomical findings and the presence or absence of ischemia is pivotal for determining the need for percutaneous coronary intervention. However, concurrently, comprehending vulnerability can greatly assist in predicting future cardiovascular events and formulating preventive strategies for individual patients. This review aims to describe the vulnerability of coronary artery plaques, primarily focusing on vulnerable plaques through pathological, morphological, and physiological viewpoints. Our review emphasizes the usefulness of coronary imaging modalities for the diagnosis of vulnerable plaques and the assessment of their rupture risk, as well as the possibility of percutaneous coronary intervention as a management strategy for plaque stabilization. Our findings show that there have been sporadic accounts of the potential of preventing cardiovascular events through early invasive treatments in patients with moderate or greater ischemia and utilizing new-generation stents to seal lipid core plaques. Thus, it is anticipated that direct intervention targeting coronary plaques, coupled with strict low-density lipoprotein-cholesterol lowering therapy, can play a vital role in suppressing future cardiovascular events and archiving zero perioperative myocardial infarction.

## Definition of vulnerable plaque

Vulnerable plaque refers to a vulnerable atheroma that has a risk of inducing cardiovascular events such as sudden cardiac death and acute coronary syndrome associated with thrombus formation. According to autopsy reports of sudden cardiac death and acute myocardial infarction, three main pathologies in coronary atherosclerotic plaque cause coronary thrombosis. “Plaque rupture” is the most frequent, accounting for 60-75%, followed by “plaque erosion” at 25-40%, and “calcified nodule” at approximately 5%^1-5)^. In a recent study that performed analysis according to intravascular modality, the pathologies occurred at a similar ratio in ST-elevation myocardial infarction (STEMI). Although the ratio of plaque erosion and calcified nodules is slightly higher in non-ST-elevation coronary syndrome, plaque rupture remains the primary cause of acute coronary syndrome^6)^.

**Figure 1:**
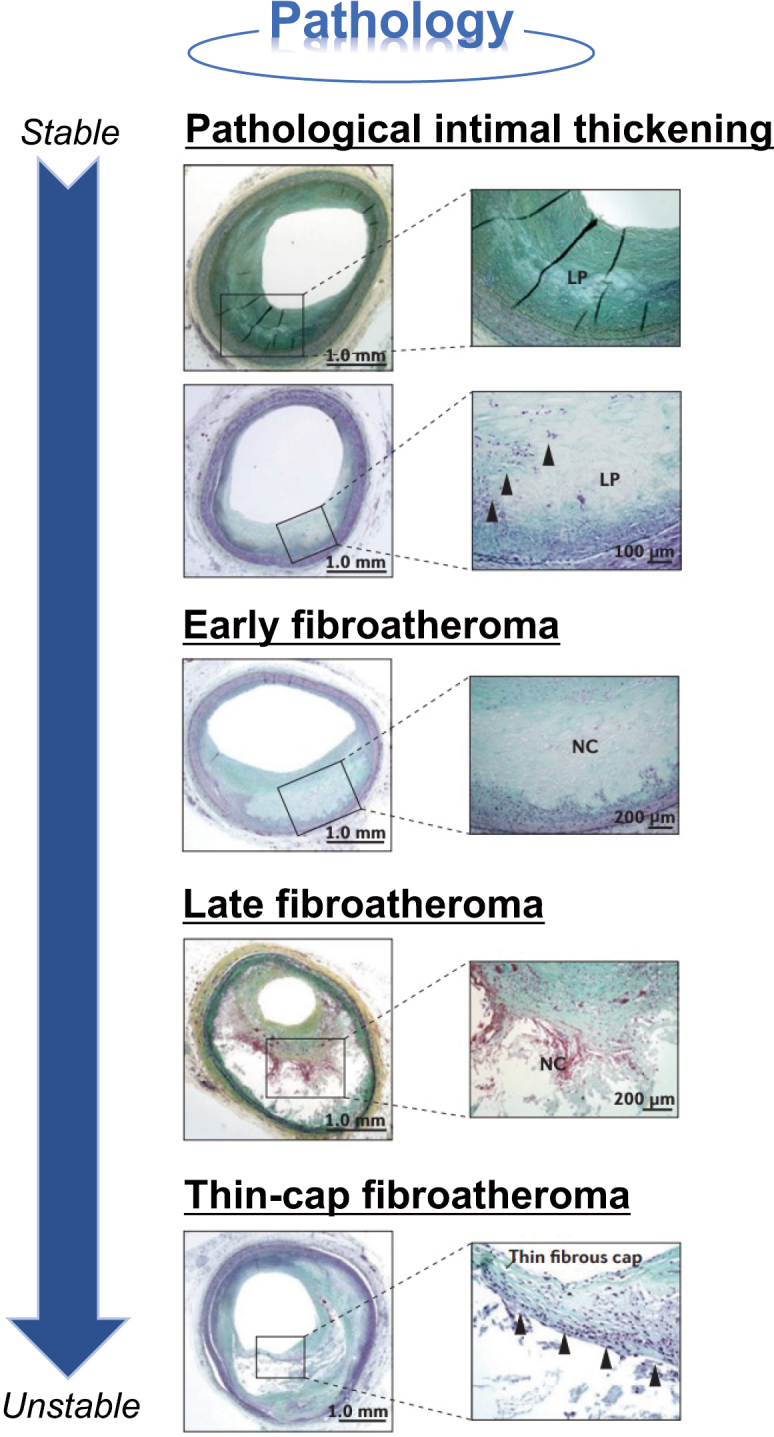
Progression to vulnerable plaque based on pathological findings The early atherosclerotic plaque progression from pathologic intimal thickening to a fibroatheroma is due to macrophage filtration (shown as ▲). Pathologic intimal thickening is characterized by the presence of LP which consist of proteoglycan with lipid insudation. The fibroatheroma has a thick fibrous cap that begins to thin over time macrophage matrix metalloproteinase release and apoptotic death of smooth muscle cells converting the fibroatheroma into a thin-cap fibroatheroma. NC, necrotic core; LP, lipid pool. Reorganized from the reference of 4) with permission from the publisher.

Since the first study of plaque rupture in an autopsy case of sudden cardiac death in 1844^7)^, there have been various discussions on the paradigm of plaque vulnerability from multiple viewpoints, such as pathological, morphological, and physiological approaches. Particularly, many studies have been conducted on pathological characteristics of plaque vulnerability since the first report. Plaque rupture is due to rupture of the fibrous cap covering the lipid core and tends to occur in vulnerable plaque with the following characteristics as shown in Figure 1: (1) large lipid core, (2) thin fibrous capsule, (3) strong infiltration of inflammatory cells such as macrophages and T lymphocytes, (4) abundant neovascularization, (5) accompanied by calcification, and (6) accompanied by positive remodeling^4, 8)^.

## Diagnosis and risk assessment of vulnerable plaque

Diagnostic imaging is crucial in understanding morphological plaque characteristics in coronary arteries. This is because approximately 70% of the lesions responsible for myocardial infarction have <50% coronary artery stenosis^9)^; identifying not only degree of coronary artery stenosis but also vulnerable plaque can lead to the prevention of future cardiovascular events.

Intravascular imaging modalities such as intravascular ultrasound (IVUS), optical coherence tomography (OCT), near-infrared spectroscopy (NIRS), and angioscopy are used in clinical practice. Pathological findings have emphasized the presence of a thin fibrous cap and an abundant lipid core as indicators of vulnerable plaques. To address the limitations of plaque tissue identification in grayscale IVUS, virtual histological IVUS (VH-IVUS), which classifies plaque properties into four types (fibrous, fibro-fatty, dense calcium, and necrotic core) and displays them using color mapping, was developed. VH-IVUS-derived thin-cap fibroatheroma (VH-TCFA) was defined according to the criteria of “atheroma volume ratio ≥40%”, “necrotic core ≥10%”, and “plaques in contact with the lumen in 3 or more sections” in the Providing Regional Observations to Study Predictors of Events in the Coronary Tree (PROSPECT) study^10)^. Its frequency was significantly higher in patients with acute coronary syndrome than in those with stable angina, and VH-TCFA was a significant predictor of cardiovascular events. Additionally, OCT has a high spatial resolution, enabling accurate measurement of fibrous cap thickness and more accurate identification of TCFAs. Notably, TCFA is defined as thin fibrous caps with a thickness of <65 μm, bearing large lipid cores and macrophage infiltration. This cutoff value is derived from the analysis of histopathological samples^1, 11)^. The presence of TCFA detected using OCT increased perioperative myocardial infarction^12)^, and was a significant predictor of cardiovascular events in the CLIMA study^13)^. This finding suggests that TCFA contributes to the development of acute coronary syndrome, concurrent perioperative myocardial infarction, and future cardiovascular event risk.

Furthermore, NIRS-IVUS, which uses NIRS for grayscale IVUS and can quantitatively evaluate the lipid core with an index called the lipid core burden index (LCBI), has been developed and applied in clinical practice. In both culprit and non-culprit lesions, lipid core plaques have been detected at a higher rate in patients with acute coronary syndrome than in those with stable angina^14)^. Additionally, the maximum LCBI within any 4-mm long segment (maxLCBI_4mm_) in culprit lesions for STEMI was 5.8 times higher than in non-culprit lesions. MaxLCBI_4mm_ ≥400 was established as the cutoff value for predicting the development of STEMI^15)^. Furthermore, in patients undergoing elective percutaneous coronary intervention (PCI) in the Coronary Assessment by Near-infrared of Atherosclerotic Rupture-prone Yellow (CANARY) trial, maxLCBI_4mm_ was significantly higher in the perioperative myocardial infarction group; however, there was no significant difference in the frequency of perioperative myocardial infarction with or without distal embolic protection in patients with maxLCBI4mm ≥600^16)^.

**Figure 2:**
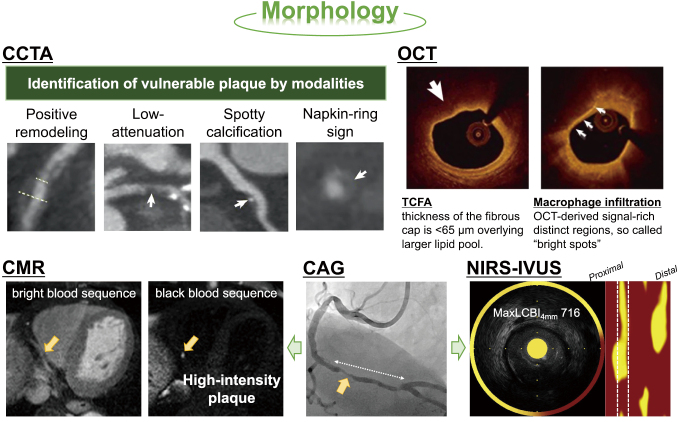
Morphological findings of vulnerable plaques on coronary modalities The findings of vulnerable coronary plaque identified by both invasive and non-invasive modalities, such as CCTA, non-contrast CMR, OCT, and NIRS-IVUS, are shown. The upper panel shows the representative vulnerable characteristics detected by CCTA. The upper right panel shows the TCFA and macrophage filtration using OCT. The lower panel shows that CMR-derived high intensity plaque, IVUS-derived attenuated plaque, and NIRS-derived large lipid-rich plaque defines as maxLCBI4mm ≥400 are measured in culprit lesion site of segment 3 indicated by the yellow arrow on CAG imaging. CAG, coronary angiography; CCTA, coronary computed tomography angiography; CMR, cardiac magnetic resonance; MaxLCBI4mm, maximum lipid core burden index within any 4-mm long segment; NIRS-IVUS, near-infrared spectroscopy and intravascular ultrasound; OCT, optical coherence tomography, TCFA, thin-cap fibroatheroma. Reorganized from the references of 17) and 19) with permission from the publisher.

Whereas, although numerous studies have been published on non-invasive examinations using coronary computed tomography angiography (CCTA), cardiac magnetic resonance (CMR), and positron emission tomography (PET), CCTA is the most practical in clinical practice. The distinctive features of vulnerable plaques identified using CCTA include (1) positive remodeling (≥1.1), (2) low-attenuated plaques (<30 Hounsfield Unit), (3) spotty calcification, and (4) napkin-ring signs. These characteristics have been reported to correlate with vulnerable plaque findings identified using VH-IVUS, OCT, and NIRS-IVUS^17, 18)^. Regarding coronary plaque detected by CMR, to visualize coronary plaques, imaging is performed using black blood method which suppresses blood flow signal, in addition, the coronary artery walls and plaques can be visualized by suppressing the fat signal around the coronary arteries. Furthermore, the coronary atherosclerosis T1-weighted characterization (CATCH) method, which combines a black blood sequence that can visualize high-intensity plaque in the coronary arteries and a bright blood sequence that can visualize the coronary artery lumen with high-brightness, has recently been developed. Notably, high-intensity plaques identified using the CATCH method were significant predictors of maxLCBI_4mm_ ≥400 and perioperative myocardial infarction, and the CATCH method was useful for vulnerable plaque detection and risk assessment of perioperative myocardial infarction before PCI^19)^. Representative findings of vulnerable plaques on both invasive and non-invasive coronary modalities were shown in Figure 2.

## Onset of clinical events in functional ischemia and vulnerable plaque

**Figure 3:**
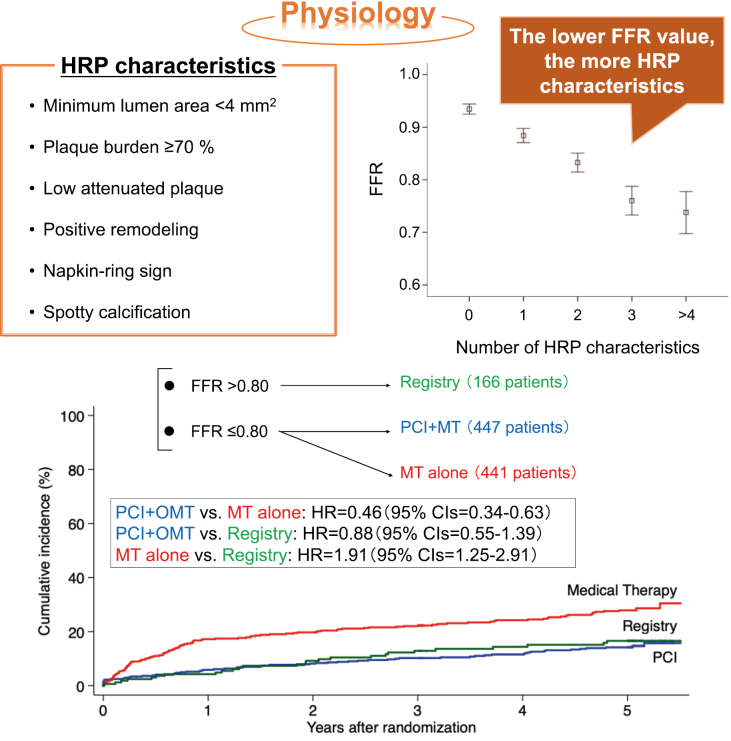
Association of physiological evaluation with vulnerable plaque characteristics and clinical outcomes The upper right panel shows the HRP characteristics items detected by CCTA, and the upper left panel shows the association between FFR value and number of its HRP characteristics. The lower panel shows the results of FAME2 trial during the follow-up period of 5 years. CCTA, coronary computed tomography angiography; FAME2, Fractional Flow Reserve versus Angiography for Multivessel Evaluation 2; FFR, fractional floe reserve; HRP, high-risk plaque; MT, medical therapy; PCI, percutaneous coronary intervention. Reorganized from the references of 21) and 22) with permission from the publisher.

One physiological evaluation of coronary arteries is fractional flow reserve (FFR), which is used as a functional evaluation for moderate stenotic lesions. The Fractional Flow Reserve Guided Percutaneous Coronary Intervention Plus Optimal Medical Therapy Versus Optimal Medical Therapy (FAME2) study compared the PCI + optimal medical therapy group and the optimal medical therapy alone group in patients with stable angina pectoris, and the cutoff value was set at FFR ≤0.80. When FFR-guided PCI was combined with optimal drug therapy, although there was no significant difference in all- cause mortality or the onset of myocardial infarction compared with the optimal drug therapy alone group, it was observed that emergency revascularization significantly decreased^20)^. A study on the association between FFR and vulnerable plaque demonstrated that the lower FFR value, the more high-risk plaque (HRP) characteristics were identified in CCTA shown in Figure 3^21)^. In addition, this study reported that the lesions with ≥3 HRP characteristics showed a significantly higher risk of vessel-orientated composite outcomes compared with those with <3 HRP characteristics in the FFR >0.80 group; however, there was no significant difference in the risk of vessel-orientated composite outcomes between ≥3 HRP characteristics versus <3 HRP characteristics in the FFR ≤0.80 group. Therefore, in FFR ischemia-positive patients, stent treatment with PCI had a certain effect in suppressing emergency revascularization and may have contributed to plaque stabilization. Furthermore, in the population that led to a diagnosis of FFR ≤0.80 ischemia in the 5-year follow-up of the FAME2 trial, although the composite endpoint (all-cause mortality, myocardial infarction, and emergency revascularization) significantly decreased in the PCI + optimal medical therapy group compared to the optimal medical therapy alone group (13.9% vs. 27.0%, *p*<0.05), 15.7% of the patients without ischemia with FFR >0.80 developed the composite endpoint and its rate was higher than the PCI + optimal drug therapy group shown in Figure 3^22)^. Even if optimal drug therapy is continued after the diagnosis of non-ischemia in FFR, the event will still occur; consequently, the presence of vulnerable plaque and residual risk in patients without ischemia is one of the challenges. Therefore, the early identification of vulnerable plaque and the reinforcement of its treatment, as well as the setting of new FFR cutoff values in the era of next- generation drug-eluting stent (DES), may help reduce the risk of cardiovascular events in patients without ischemia.

## Possibility of PCI for vulnerable plaque

Regarding PCI in patients with stable angina pectoris, many studies have indicated that PCI does not enhance prognosis, although it is expected to improve angina symptoms, and the Clinical Outcomes Utilizing Revascularization and Aggressive Drug Evaluation (COURAGE) trial is often regarded as a representative study^23)^. The COURAGE trial compared the PCI + medical therapy group with a medical therapy group and defined a composite endpoint of all-cause mortality and nonfatal myocardial infarction. No significant difference was observed in the composite endpoint during the follow-up period of 4.6 years between the PCI + medical and medical therapy groups (18.5% vs. 19.0%, *p*=0.62). However, the COURAGE trial had limitations, including the fact that most of the stents used were bare-metal stent (BMS), and high-risk patients were excluded. Nevertheless, the International Study of Comparative Health Effectiveness with Medical and Invasive Approaches (ISCHEMIA) trial published in 2020 eventually included 5,179 patients with stable angina with moderate or greater ischemia. A comparison was made between an early invasive strategy group (PCI [87% 2^nd^ generation DES usage] or coronary artery bypass grafting) and a conservative strategy group^24)^. During the median follow-up period of 3.2 years, no significant difference was observed between the two groups regarding the composite endpoint of cardiovascular mortality, myocardial infarction, unstable angina, heart failure, resuscitated cardiac arrest, or all-cause mortality. However, although the event risk was low in the conservative treatment strategy group in the first half, that tended to decrease gradually in the early invasive strategy group in the second half. In ISCHEMIA-EXTEND trial, which was started as an even longer-term prognostic evaluation, the cardiovascular mortality rate decreased in the early invasive strategy group during the follow-up period of 7 years^25)^. The evolution of DES may also be cited as a factor contributing to the increasing disparity between the two groups.

DES enabled a notable reduction in restenosis compared to BMS. Nonetheless, certain problems emerged, including (1) thrombotic risk due to inadequate coverage of the neointima, (2) poor long-term results of stenting in lipid-rich lesions, and (3) late events due to chronic inflammation caused by polymers in 1^st^ generation DES^26, 27)^. Therefore, 2^nd^ generation DES has improved design, drug concentration, and polymer to overcome such weaknesses, and are currently the most widely used devices. Several reports of stent performance for vulnerable plaque lesions with abundant lipid cores in such next-generation DES shall be presented.

The Chemometric Observations of Lipid Core Plaques of Interest in Native Coronary Arteries Registry) COLOR trial evaluated NIRS-detected lipid core plaques in culprit lesions and clinical outcomes (major cardiovascular events (MACEs): cardiovascular mortality, myocardial infarction, stent thrombosis, and unplanned revascularization or readmission for progressive or unstable angina) in patients with coronary artery disease treated with DES (approximately 80% are 2^nd^ generation DES)^28)^. MACEs accounted for 18.0% overall and 8.3% for the culprit lesion only. There was no significant difference in maxLCBI_4mm_ using NIRS and minimum lumen area and plaque burden using IVUS with or without an event related to the culprit lesion. Moreover, no significant difference was observed in MACEs when maxLCBI_4mm_ was divided into three groups and compared with each other (maxLCBI_4mm_ = 0-196, 197-420, 421-1,000). Additionally, a Revelation of Pathophysiological Phenotypes of Vulnerable Lipid-Rich Plaque (REASSURE)-NIRS trial evaluated the effect of a residual maxLCBI4mm after stent placement on lesion-derived (cardiovascular mortality, nonfatal target lesion-related myocardial infarction, and target lesion revascularization) and patient-derived (all- cause mortality, nonfatal myocardial infarction, and unplanned revascularization) clinical outcomes^29)^. The median residual maxLCBI_4mm_ was 183, and 16% of all lesions had a residual maxLCBI_4mm_ >400. The subjects were divided into three groups according to the tertile of residual maxLCBI_4mm_ (residual maxLCBI_4mm_ = 0-123, 124-270, 271-799). There was no significant difference in the occurrence of events among the three groups, and residual maxLCBI_4mm_ did not emerge as a significant predictor of those events. In short, no difference was observed in lesion-derived outcomes depending on the residual maxLCBI_4mm_ value due to 2^nd^ generation DES usage. Compared with the 1^st^ generation DES, 2^nd^ generation DES had a greater strut coverage with less inflammation, less fibrin deposition, and less late and very late stent thrombosis^30)^. In addition, the 2^nd^ generation DES was associated with a significantly reduced risk of long-term target lesion failure than the 1^st^ generation DES, although there were no significant differences in cardiac death and myocardial infarction^31)^. Thus, 2^nd^ generation DES could suppress arteriosclerosis and contribute to reducing events only in stented lesion site. These findings at least suggest that the lipid core in the culprit lesion might be sealed.

A study on the sealing effect of stents demonstrated that everolimus-eluting stent placement significantly reduced the number of macrophages involved in vulnerable plaque formation in an animal study^32)^. Recently, the PROSPECT ABSORB trial was reported, which evaluated the effect of bioabsorbable vascular scaffold (BVS) for non-stenotic lesions (moderate stenosis and functional ischemia negative) with plaque burden ≥65 % assessed using IVUS, and the subjects were divided into two groups (BVS + guideline-directed medical therapy [GDMT] and GDMT alone)^33)^. This study evaluated IVUS-derived minimum lumen area at the follow-up period, the primary safety endpoint was target lesion failure (cardiovascular death, target vessel-related myocardial infarction, or clinically driven target lesion revascularization), and secondary clinical effectiveness endpoint was lesion- related MACEs (cardiovascular death, myocardial infarction, unstable angina, or progressive angina). During the median follow-up period of 4.1 years, the minimum lumen area was 6.9 ± 2.6 mm^2^ vs. 3.0 ± 1.0 mm^2^ in the BVS + GDMT and GDMT alone groups, respectively (least square means difference: 3.9 mm^2^, *p*<0.05). Although no significant differences were observed in primary and secondary endpoints between the two groups, secondary endpoint rate tended to be lower in the BVS + GDMT group (4.3% vs. 10.7%, *p*=0.12). Thus, prophylactic plaque stabilization of BVS against high-risk plaques with non-ischemic moderate stenosis was confirmed. However, prophylactic PCI for FFR- negative vulnerable plaque relies on very small effects, and there are certain limitations due to the enormous sample size required^34)^.

## Achieving zero perioperative myocardial infarction

As stated above, the characteristic finding of vulnerable plaque identified using preoperative and intraoperative modalities is a strong predictor of perioperative myocardial infarction. Plaque stabilization is considered the most critical factor in preventing perioperative myocardial infarction, and expectations are high for plaque sealing with stents. However, preventing iatrogenic perioperative myocardial infarction is considered a prerequisite for plaque-sealing treatment, even though plaque rupture has not occurred. Moreover, statin, ezetimibe, or proprotein convertase subtilisin/kexin type 9 (PCSK9) inhibitor combination therapy has been reported to stabilize and regress coronary artery plaques, and low-density lipoprotein-cholesterol (LDL-C) lowering therapy is essential for the prevention of perioperative myocardial infarction^35-37)^. The Progression of Atherosclerotic Plaque Determined by Computed Tomographic Angiography Imaging (PRADIGM) trial reported that statins contributed to increased plaque calcification and decreased characteristic findings of high-risk plaques, slowing the progression of overall coronary atherosclerosis^38)^. Although the period of plaque stabilization has not been clarified, a case report has reported changes over time in LDL-C levels due to strict LDL-C lowering therapy and maxLCBI_4mm_ at non-culprit lesion segment using NIRS^39)^. In addition to statin, a PCSK9 inhibitor was used in combination, and LDL-C decreased rapidly over several months and remained at the 10 mg/dL level. Meanwhile, the maxLCBI_4mm_ at the non-culprit lesion site of left main tract gradually decreased from 422 to 109 over 2 years, quantitatively demonstrating favorable results for lipid core plaques. Therefore, based on the target LDL-C levels for primary and secondary prevention of coronary artery disease in Japanese guidelines^40)^, aggressive lipid management from an early stage expecting long-term benefits may contribute to the suppression of perioperative complications. Prevention and treatment of comorbidities such as hypertension, diabetes, and chronic kidney disease, as well as guidance on diet, exercise, and smoking cessation, are equally crucial.

## Conclusion

In this article, we described the vulnerability of coronary artery plaques (mainly vulnerable plaque) from pathological, morphological, and physiological viewpoints. In our daily clinical practice, it is crucial to evaluate anatomical findings and the presence or absence of ischemia in determining the indication for PCI. Simultaneously, understanding and grasping plaque vulnerability will be of great help in predicting future cardiovascular events and in developing preventive strategies in individual patients. Recently, there have been sporadic reports on myocardial infarction prevention using early invasive treatment and the sealing effect of lipid core plaques by the deployment of new-generation stents. In the future, it is expected that direct plaque intervention, in addition to strict LDL-C lowering therapy, may lead to prevention of plaque rapture and suppression of cardiovascular event, not only by identifying vulnerable plaques but also by considering active therapeutic intervention.

## Funding

The authors declare no funding.

## Author contributions

TF and TD wrote and reviewed the manuscript. All authors read and approved the final manuscript.

## Conflicts of interest statement

The authors report no relationships that could be construed as a conflict of interest.

## References

[1] Virmani R, Kolodgie FD, Burke AP, Farb A, Schwartz SM: Lessons from sudden coronary death: a comprehensive morphological classification scheme for atherosclerotic lesions. Arterioscler Thromb Vasc Biol, 2000; 20: 1262-1275. 10807742 10.1161/01.atv.20.5.1262

[2] Burke AP, Farb A, Malcom GT, Liang YH, Smialek J, Virmani R: Coronary risk factors and plaque morphology in men with coronary disease who died suddenly. N Engl J Med, 1997; 336: 1276-1282. 9113930 10.1056/NEJM199705013361802

[3] Farb A, Burke AP, Tang AL, et al: Coronary plaque erosion without rupture into a lipid core. A frequent cause of coronary thrombosis in sudden coronary death. Circulation, 1996; 93: 1354-1363. 8641024 10.1161/01.cir.93.7.1354

[4] Yahagi K, Kolodgie FD, Otsuka F, et al: Pathophysiology of native coronary, vein graft, and in-stent atherosclerosis. Nat Rev Cardiol, 2016; 13: 79-98. 26503410 10.1038/nrcardio.2015.164

[5] Torii S, Sato Y, Otsuka F, et al: Eruptive calcified nodules as a potential mechanism of acute coronary thrombosis and sudden death. J Am Coll Cardiol, 2021; 77: 1599-1611. 33795033 10.1016/j.jacc.2021.02.016

[6] Yamamoto E, Yonetsu T, Kakuta T, et al: Clinical and laboratory predictors for plaque erosion in patients with acute coronary syndromes. J Am Heart Assoc, 2019; 8: e012322. 31640466 10.1161/JAHA.119.012322 PMC6898801

[7] Finn AV, Nakano M, Narula J, Kolodgie FD, Virmani R: Concept of vulnerable/unstable plaque. Arterioscler Thromb Vasc Biol, 2010; 30: 1282-1292. 20554950 10.1161/ATVBAHA.108.179739

[8] Falk E: Pathogenesis of atherosclerosis. J Am Coll Cardiol, 2006; 47(8 Suppl): C7-12. 10.1016/j.jacc.2005.09.068 16631513

[9] Falk E, Shah PK, Fuster V: Coronary plaque disruption. Circulation, 1995; 92: 657-671. 7634481 10.1161/01.cir.92.3.657

[10] Stone GW, Maehara A, Lansky AJ, et al: A prospective natural-history study of coronary atherosclerosis. N Engl J Med, 2011; 364: 226-235. 21247313 10.1056/NEJMoa1002358

[11] Tearney GJ, Regar E, Akasaka T, et al: Consensus standards for acquisition, measurement, and reporting of intravascular optical coherence tomography studies: a report from the International Working Group for Intravascular Optical Coherence Tomography Standardization and Validation. J Am Coll Cardiol, 2012; 59: 1058-1072. 22421299 10.1016/j.jacc.2011.09.079

[12] Kini AS, Motoyama S, Vengrenyuk Y, et al: Multimodality intravascular imaging to predict periprocedural myocardial infarction during percutaneous coronary intervention. JACC Cardiovasc Interv, 2015; 8: 937-945. 26088511 10.1016/j.jcin.2015.03.016

[13] Prati F, Romagnoli E, Gatto L, et al: Relationship between coronary plaque morphology of the left anterior descending artery and 12 months clinical outcome: the CLIMA study. Eur Heart J, 2020; 41: 383-391. 31504405 10.1093/eurheartj/ehz520

[14] Madder RD, Smith JL, Dixon SR, Goldstein JA: Composition of target lesions by near-infrared spectroscopy in patients with acute coronary syndrome versus stable angina. Circ Cardiovasc Interv, 2012; 5: 55-61. 22253357 10.1161/CIRCINTERVENTIONS.111.963934

[15] Madder RD, Goldstein JA, Madden SP, et al: Detection by near-infrared spectroscopy of large lipid core plaques at culprit sites in patients with acute ST-segment elevation myocardial infarction. JACC Cardiovasc Interv, 2013; 6: 838-846. 23871513 10.1016/j.jcin.2013.04.012

[16] Stone GW, Maehara A, Muller JE, et al: Plaque characterization to inform the prediction and prevention of periprocedural myocardial infarction during percutaneous coronary intervention: The CANARY trial (coronary assessment by near-infrared of atherosclerotic rupture-prone yellow). JACC Cardiovasc Interv, 2015; 8: 927-936. 26003018 10.1016/j.jcin.2015.01.032

[17] Föllmer B, Williams MC, Dey Damini, et al: Roadmap on the use of artificial intelligence for imaging of vulnerable atherosclerotic plaque in coronary arteries. Nat Rev Cardiol, 2024; 21: 51-64. 37464183 10.1038/s41569-023-00900-3

[18] Bom MJ, van der Heijden DJ, Kedhi E, et al: Early detection and treatment of the vulnerable coronary plaque: can we prevent acute coronary syndromes? Circ Cardiovasc Imaging, 2017; 10: e005973. 28483945 10.1161/CIRCIMAGING.116.005973

[19] Fukase T, Dohi T, Fujimoto S, et al: Relationship between coronary high-intensity plaques on T1-weighted imaging by cardiovascular magnetic resonance and vulnerable plaque features by near-infrared spectroscopy and intravascular ultrasound: a prospective cohort study. J Cardiovasc Magn Reson 2023; 25: 4. 36710360 10.1186/s12968-023-00916-1 PMC9885661

[20] De Bruyne B, Pijls NH, Kalesan B, et al: Fractional flow reserve-guided PCI versus medical therapy in stable coronary disease. N Engl J Med, 2012; 367: 991-1001. 22924638 10.1056/NEJMoa1205361

[21] Lee JM, Choi KH, Koo BK, et al: Prognostic implications of plaque characteristics and stenosis severity in patients with coronary artery disease. J Am Coll Cardiol, 2019; 73: 2413-2424. 31097161 10.1016/j.jacc.2019.02.060

[22] Xaplanteris P, Fournier S, Pijls NHJ, et al: Five-year outcomes with PCI guided by fractional flow reserve. N Engl J Med, 2018; 379: 250-259. 29785878 10.1056/NEJMoa1803538

[23] Boden WE, O’Rourke RA, Teo KK, et al: Optimal medical therapy with or without PCI for stable coronary disease. N Engl J Med, 2007; 356: 1503-1516. 17387127 10.1056/NEJMoa070829

[24] Maron DJ, Hochman JS, Reynolds HR, et al: Initial invasive or conservative strategy for stable coronary disease. N Engl J Med, 2020; 382: 1395-1407. 32227755 10.1056/NEJMoa1915922 PMC7263833

[25] Bradley SM, Gluckman TJ: If the Fates Allow: The zero-sum game of ISCHEMIA-EXTEND. Circulation, 2023; 147: 20-22. 36335920 10.1161/CIRCULATIONAHA.122.063033

[26] Daemen J, Wenaweser P, Tsuchida K, et al: Early and late coronary stent thrombosis of sirolimus-eluting and paclitaxel-eluting stents in routine clinical practice: data from a large two-institutional cohort study. Lancet, 2007; 369: 667-678. 17321312 10.1016/S0140-6736(07)60314-6

[27] Finn AV, Joner M, Nakazawa G, et al: Pathological correlates of late drug-eluting stent thrombosis: strut coverage as a marker of endothelialization. Circulation, 2007; 115: 2435-2441. 17438147 10.1161/CIRCULATIONAHA.107.693739

[28] Yamamoto MH, Maehara A, Stone GW, et al: 2-Year outcomes after stenting of lipid-rich and nonrich coronary plaques. J Am Coll Cardiol, 2020; 75: 1371-1382. 32216905 10.1016/j.jacc.2020.01.044

[29] Murai K, Kataoka Y, Nicholls SJ, et al: The residual lipid-rich coronary atheroma behind the implanted newer-generation drug-eluting stent and future stent-related event risks. Can J Cardiol, 2022; 38: 1504-1515. 35840020 10.1016/j.cjca.2022.07.004

[30] Otsuka F, Vorpahl M, Nakano M, et al: Pathology of second-generation everolimus-eluting stents versus first-generation sirolimus- and paclitaxel-eluting stents in humans. Circulation, 2014; 129: 211-223. 24163064 10.1161/CIRCULATIONAHA.113.001790 PMC3915802

[31] Choi KH, Song YB, Lee JM, et al: Differential Long-Term Effects of First- and Second-Generation DES in Patients With Bifurcation Lesions Undergoing PCI. JACC Asia, 2021; 1: 68-79. 36338362 10.1016/j.jacasi.2021.04.006 PMC9627880

[32] Verheye S, Roth L, De Meyer I, et al: Cryotherapy increases features of plaque stability in atherosclerotic rabbits. EuroIntervention, 2016; 12: 748-756. 26448576 10.4244/EIJY15M10_02

[33] Stone GW, Maehara A, Ali ZA, et al: Percutaneous coronary intervention for vulnerable coronary atherosclerotic plaque. J Am Coll Cardiol, 2020; 76: 2289-2301. 33069847 10.1016/j.jacc.2020.09.547

[34] Zimmermann FM, Pijls NHJ, Gould KL, Johnson NP: Stenting “vulnerable” but fractional flow reserve-negative lesions: potential statistical limitations of ongoing and future trials. JACC Cardiovasc Interv, 2021; 14: 461-467. 33602443 10.1016/j.jcin.2020.05.036

[35] Okazaki S, Yokoyama T, Miyauchi K, et al: Early statin treatment in patients with acute coronary syndrome: demonstration of the beneficial effect on atherosclerotic lesions by serial volumetric intravascular ultrasound analysis during half a year after coronary event: the ESTABLISH Study. Circulation, 2004; 110: 1061-1068. 15326073 10.1161/01.CIR.0000140261.58966.A4

[36] Tsujita K, Sugiyama S, Sumida H, et al: Impact of dual lipid-lowering strategy with ezetimibe and atorvastatin on coronary plaque regression in patients with percutaneous coronary intervention: The multicenter randomized controlled PRECISE-IVUS trial. J Am Coll Cardiol, 2015; 66: 495-507. 26227186 10.1016/j.jacc.2015.05.065

[37] Ota H, Omori H, Kawasaki M, Hirakawa A, Matsuo H: Clinical impact of PCSK9 inhibitor on stabilization and regression of lipid-rich coronary plaques: a near-infrared spectroscopy study. Eur Heart J Cardiovasc Imaging, 2022; 23: 217-228. 33637979 10.1093/ehjci/jeab034

[38] Lee SE, Chang HJ, Sung JM, et al: Effects of statins on coronary atherosclerotic plaques: The PARADIGM Study. JACC Cardiovasc Imaging, 2018; 11: 1475-1484. 29909109 10.1016/j.jcmg.2018.04.015

[39] Takahashi N, Dohi T, Endo H, Okazaki S: Stepwise regression of non-culprit lipid-rich plaque observed using serial near-infrared spectroscopy-intravascular ultrasound and optical coherence tomographic measurements after aggressive cholesterol-lowering treatment: a case report. Eur Heart J Case Rep, 2021; 5: ytab095. 34113771 10.1093/ehjcr/ytab095 PMC8186931

[40] Okamura T, Tsukamoto K, Arai H, et al: Japan Atherosclerosis Society (JAS) Guidelines for Prevention of Atherosclerotic Cardiovascular Diseases 2022. J Atheroscler Thromb, 2023. Online ahead of print. 10.5551/jat.GL2022 PMC11150976 38123343

